# Occlusion par hernie interne transmesenterique congénitale: à propos de deux cas

**DOI:** 10.11604/pamj.2017.27.131.12203

**Published:** 2017-06-20

**Authors:** Souleymane Ouedraogo, Salam Ouedraogo, Jean Luc Kambire, Maurice Zida, Adama Sanou

**Affiliations:** 1 Centre Hospitalier Universitaire d’Ouahigouya, Ouahigouya, Burkina Faso; 2 Centre Hospitalier Universitaire Yalgado, Ouedraogo, Burkina Faso

**Keywords:** Hernie interne, hernie transmésenterique, occlusion intestinale aiguë, Internal hernia, transmesenteric hernia, acute intestinal obstruction

## Abstract

La hernie interne à travers un défect du mésentère ou hernie transmésentérique est une cause rare d'occlusion intestinale aiguë. Son diagnostic est le plus souvent réalisé en per opératoire. La connaissance de ses particularités cliniques permet d'envisager le diagnostic préopératoire. Nous présentons 2 cas d'occlusion intestinale aiguë causée par une hernie transmésentérique congénitale chez 2 adultes. Nous discutons des particularités cliniques de cette forme rare de hernie interne.

## Abstract (en)

Internal hernia due to mesenteric defect or transmesenteric hernia is a rare cause of acute intestinal obstruction. Its diagnosis is most often done during surgery. The knowledge of its clinical peculiarities allows the preoperative diagnosis. We here report 2 cases of acute intestinal obstruction secondary to congenital transmesenteric hernia in two adult patients. This study aims to highlight the clinical peculiarities of this rare form of internal hernia.

## Introduction

Les occlusions intestinales aiguës par hernie interne sont rares [1]. Leur diagnostic est le plus souvent réalisé en peropératoire [2]. Les formes anatomiques de hernie interne sont nombreuses, certaines étant très rarement rapportées. Cependant, la connaissance des différentes variétés de hernies internes est fondamentale pour envisager un diagnostic préopératoire. La hernie transmésenterique congénitale de l'adulte est une forme rare de hernie interne [3, 4]. Nous présentons deux cas d'occlusion intestinale aiguë par hernie interne transmésenterique congénitale traités dans le service de chirurgie générale de l'hôpital régional de Tenkodogo, au Burkina Faso, afin de contribuer à la connaissance des particularités cliniques de cette entité.

## Patient et observation

**Figure 1:**
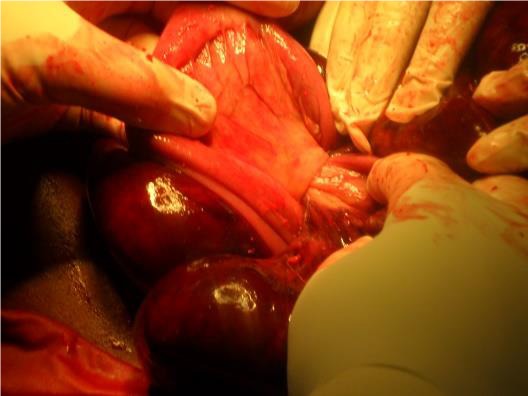
Vue opératoire d’une hernie transmésentérique

**Figure 2:**
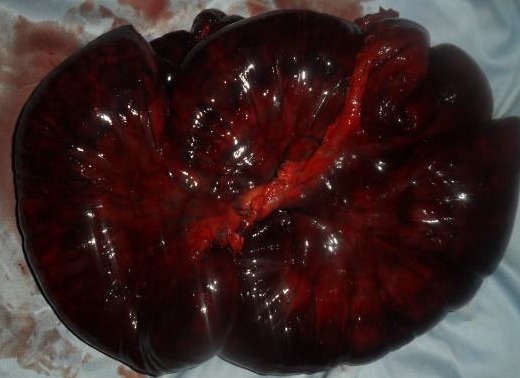
Segment intestinal nécrosé

**Observation 1:** Un homme de 56 ans a été admis dans le service de chirurgie générale de l'hôpital régional de Tenkodogo, au Burkina Faso pour douleurs abdominales diffuses, vomissements alimentaires et arrêt des matières et des gaz. Cette symptomatologie évoluait depuis 48 heures. L'interrogatoire note la survenue régulière de crises similaires ayant cédé au bout de quelques heures. Il n'a pas été retrouvé d'antécédent de chirurgie abdominale, ni de traumatisme abdominal. L'examen physique a confirmé la présence d'un syndrome occlusif avec distension abdominale et météorisme. Les orifices herniaires pariétaux étaient libres. Le reste de l'examen physique était normal. La radiographie de l'abdomen sans préparation a noté des niveaux hydro-aériques de type grêlique. La tomodensitométrie n'était pas disponible en urgence. L'hémogramme, la glycémie et la créatininémie étaient dans les limites de la normale. Le diagnostic d'occlusion intestinale aiguë a été retenu. Une laparotomie a été indiquée en urgence. L'incision a été une médiane. En per opératoire, nous avons noté une incarcération d'anses iléales à travers un défect d'environ 6 centimètres de long, situé au niveau du mésentère (Figure 1). L'iléon incarcéré était partiellement nécrosé. Il s'agissait d'une occlusion intestinale aiguë par hernie interne transmésenterique. En absence d'antécédent de chirurgie ou de traumatisme abdominal, l'origine congénitale de la hernie a été retenue. Le traitement a consisté à une résection de l'anse nécrosée (Figure 2), suivie d'une anastomose iléo-iléale. La brèche mésentérique a été refermée. L'exploration du reste de la cavité péritonéale n'a pas noté d'autres anomalies. Les suites opératoires ont été simples. La sortie de l'hôpital a été autorisée au septième jour post opératoire.

**Figure 3:**
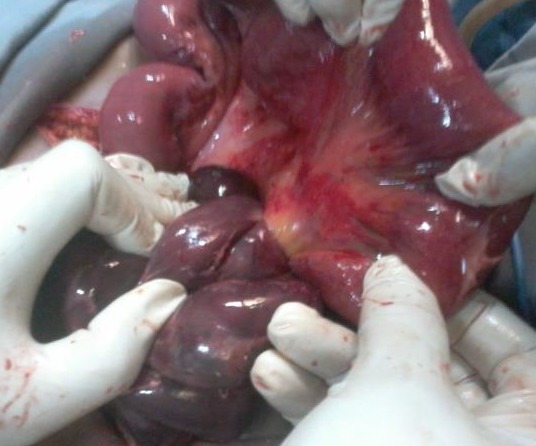
Hernie transmésenterique avec nécrose iléale

**Observation 2:** Une patiente de 35 ans, séropositive au VIH et sous traitement antirétroviral depuis 9 mois, a été admise en urgence pour douleurs abdominales diffuses de survenue brutale. Aucun antécédent de chirurgie abdominale ou de traumatisme abdominal n'a été retrouvé à l'interrogatoire. L'examen physique a retrouvé une défense abdominale diffuse. Le diagnostic de péritonite aiguë généralisée a été évoqué. La radiographie de l'abdomen sans préparation a révélé la présence de niveaux hydro-aériques de type grêlique. L'hémogramme était normal en dehors d'un décompte leucocytaire à 8 800 éléments par millimètre cube. La créatininémie était normale. Le diagnostic préopératoire évoqué était une péritonite aiguë généralisée. Une laparotomie a été donc indiquée en urgence. Une anesthésie générale avec intubation orotrachéale a été conduite et la voie d'abord a été une médiane. A l'ouverture, la cavité péritonéale était le siège d'un épanchement sanguinolent. L'exploration a noté l'incarcération d'un segment de l'iléon dans un défect du mésentère (Figure 3). L'iléon incarcéré était nécrosé sur 80 centimètres environ. Le traitement a consisté à une résection de l'iléon nécrosé, suivie d'une anastomose dans le même temps. Les suites opératoires ont été compliquées d'une septicémie, traitée avec succès par des antibiotiques. La patiente a été sortie au 12 ^ème^ jour post opératoire.

## Discussion

Les hernies internes constituent une cause rare d'occlusion intestinale aiguë [1, 2]. Elles représentent environ 5% de l'ensemble des causes d'occlusion intestinale aiguë [1]. Leur diagnostic est généralement fait en peropératoire [2, 5]. Cependant, avec le développement de l'imagerie médicale, et en particulier du scanner et de l'imagerie par résonnance magnétique, le diagnostic préopératoire est de nos jours possible. De ce fait, la connaissance des différentes variétés de hernies internes présente un intérêt de premier plan. En effet, le diagnostic d'une occlusion intestinale par hernie interne implique la parfaite connaissance de la variété anatomique en cause [6]. Plusieurs formes anatomiques de hernies internes ont été rapportées. Les hernies transmésenteriques sont définies par une protrusion d'un viscère intra abdominal à travers un défect situé au niveau du mésentère ou du mésocôlon [4]. Cette variété représente moins de 5 % des hernies internes [1, 7]. Notre premier patient a été admis pour syndrome occlusif. La particularité est l'existence, dans les antécédents, de nombreuses crises similaires ayant cédé spontanément au bout de quelques heures. Ce qui suggère la survenue d'étranglements herniaires spontanément réduites. Devant une occlusion du sujet jeune sans antécédent de chirurgie ou de traumatisme abdominal, le diagnostic de hernie interne peut être évoqué, surtout lorsque l'interrogatoire note des épisodes de sub occlusion avec rémission spontanée. Malgré ces particularités cliniques, le diagnostic clinique pré opératoire des hernies transmésenteriques demeure difficile [3]. Cependant, l'apport du scanner et de l'imagerie par résonnance magnétique est considérable. Quelques diagnostics scanographiques ont pu être réalisés [2]. Nos patients ne présentaient aucun antécédent de chirurgie abdominale. L'hypothèse congénitale peut donc être retenue. L'occlusion par hernie transmésenterique survient majoritairement chez l'enfant [8]. Sa survenue chez l'adulte est rarissime. La nécrose intestinale a été notée dans nos 2 observations. Ceci serait dû au diagnostic tardif.

## Conclusion

La hernie transmésenterique est une cause rare, mais possible d'occlusion intestinale aiguë chez l'adulte. La présence d'épisodes de sub-occlusion spontanément réduites peut être un argument important du diagnostic. Le diagnostic tardif peut occasionner des complications à type de nécrose d'anse.

## Conflits d’intérêts

Les auteurs ne déclarent aucun conflits d'intérêts.
